# Resection of a cardiac lipoma and reconstruction wall of right atrium: A case report

**DOI:** 10.1097/MD.0000000000041329

**Published:** 2025-01-31

**Authors:** Zhi Wen, Zheng Wang, Jian Guo, Changxue Wu

**Affiliations:** a Department of Cardiothoracic and Vascular Surgery, Deyang People’s Hospital, Deyang, Sichuan, China.

**Keywords:** cardiac lipoma, case report, reconstruction, right atrium, surgery

## Abstract

**Rationale:**

Cardiac lipomas are known to cause functional disturbances and symptoms by compressing adjacent tissues or organs, leading to potential complications such as dyspnea, palpitations, and cardiac arrhythmias. We report a case of a 52-year-old female with a large, well-circumscribed lipoma in the right atrium. This rare condition required a comprehensive diagnostic approach and therapeutic strategy for effective management.

**Patient concerns:**

A 52-year-old female patient presented to the hospital with complaints of palpitations and fatigue lasting for 1 month, accompanied by the recent discovery of a cardiac mass via echocardiography over the past 2 days. In addition, she reported occasional episodes of a dry cough.

**Diagnoses:**

Both echocardiography and cardiac computed tomography imaging revealed an isoechoic mass within the right atrium, characterized by a regular shape and close attachment to the right atrial wall, displaying noticeable mobility. Histopathological analysis following surgical intervention confirmed that the tumor was predominantly comprised of adipocytes.

**Interventions:**

The patient underwent successful resection of the right atrial lipoma, followed by reconstruction of right atrium using a bovine pericardial patch under extracorporeal circulation with a beating heart.

**Outcomes:**

Postoperative recovery was complete, with resolution of symptoms including palpitations and fatigue. A follow-up echocardiogram on the 66th day postsurgery confirmed the absence of any residual tumor.

**Lessons:**

Patients with small lipoma often remain asymptomatic. However, large or rapidly progressing tumors may elicit symptoms such as chest pain, dyspnea, and palpitations. For asymptomatic patients with small tumors, regular observation and follow-up are typically advised to monitor tumor growth and the emergence of symptoms. Conversely, patients with large tumors or overt symptoms should be recommended for prompt surgical intervention. In this case, preoperative anatomical evaluation for the lipoma involves the right atrial free wall, which is crucial to prevent excessive resection, damage to the lateral bundle branch, and subsequent postoperative cardiac dysfunction or arrhythmia, as exemplified in this patient.

## 1. Introduction

Primary cardiac tumors are rare, with reported incidence rate ranging from 0.0017% to 0.28%.^[1]^ Among these, cardiac lipomas account for ≈8.4% of cases,^[2]^ predominantly found in the subendocardial area (≈50%) and frequently located in the right atrium and left ventricle.^[3]^ Cardiac lipomas can occur at any age, with a higher prevalence is observed in individuals aged 40 to 60 years, with no gender preference.^[4–6]^ Despite their relatively common occurrence, large-scale clinical data on cardiac lipomas are scarce. Most available literature consists of individual case reports from both domestic and international sources. The aim of this report was to contribute to the understanding of cardiac lipoma management by describing a unique case involving the resection of a giant right atrial lipoma.

## 2. Case report

**Figure 1.**
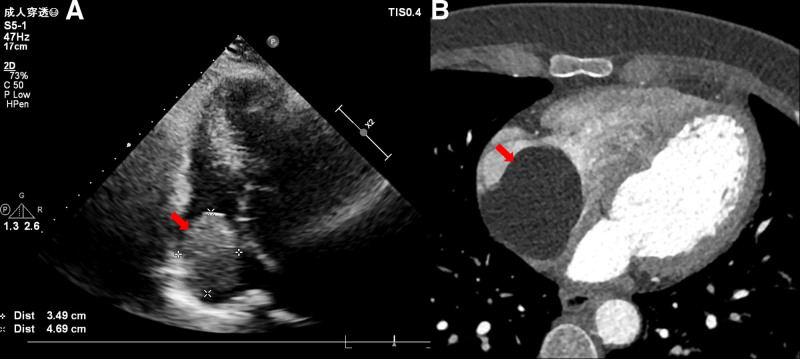
The images show a mass in the right atrium. (A) Echocardiography transcardial apex section: a 4.69 × 3.49 cm^2^ mass in the right atrium, with a regular shape, no lobulation, attached to free wall, and mobile (red arrow). (B) Cardiac CTA perfusion imaging demonstrated a homogenous low–density mass (−114 HU) in the right atrium, measuring 6.0 × 4.1 cm^2^, with a clear boundary. No enhancement was observed on the enhanced scan (red arrow). CTA = computed tomography angiography.

**Figure 2.**
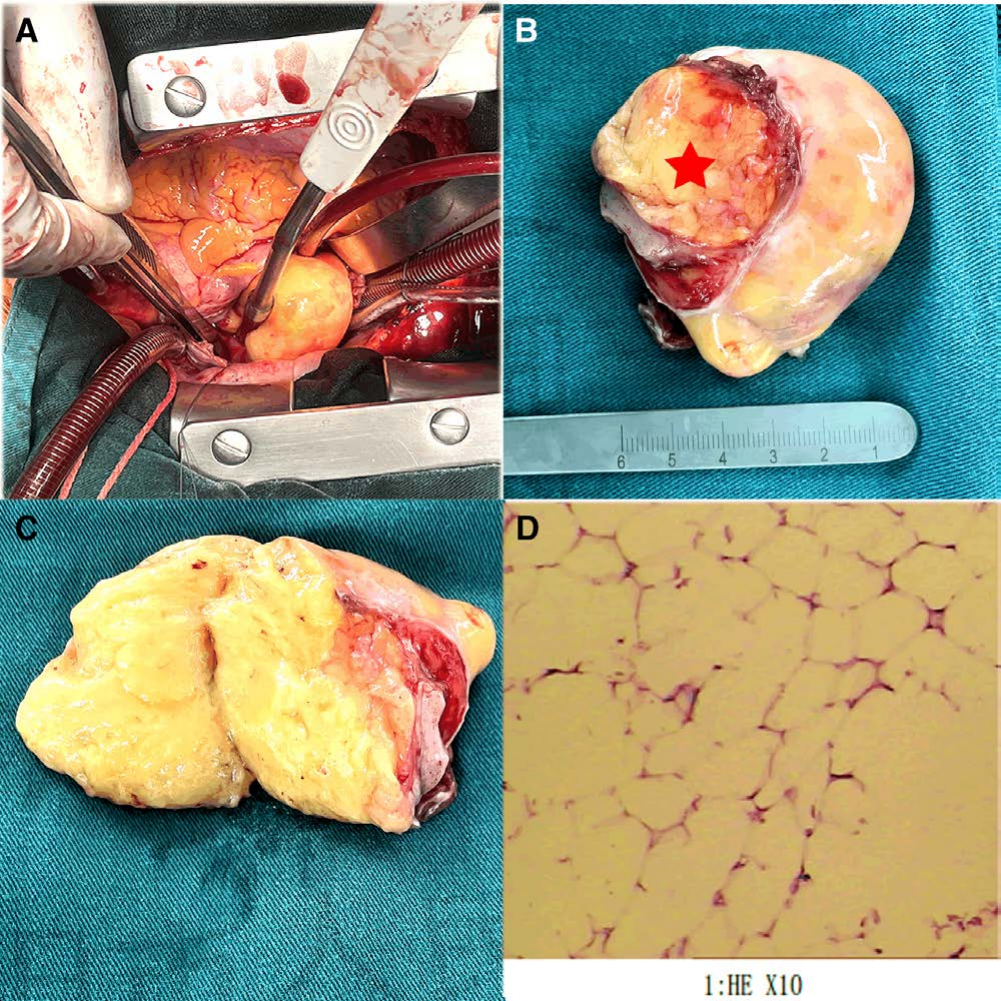
The images show the mass observed during and after surgery. (A) A yellow, soft, and smooth tumor located in the right atrium. (B) In vitro: a wide-based yellow tumor is shown (marked with a red pentagram). (C) Sectioning the tumor reveals adipose tissue with a uniform texture. (D) HE staining (×10) demonstrated numerous adipocytes. HE = hematoxylin and eosin.

A 52-year-old female was admitted to the hospital due to persistent palpitations and fatigue lasting for 1 month, accompanied by a recent detection of a cardiac mass via echocardiography 2 days prior. In addition, she experienced occasional instances of an irritating dry cough. Physical examination revealed a normal body temperature (36.3°C), pulse rate (60 beats/min) and blood pressure (96/68 mm Hg). Notably, no heart murmurs were auscultated. Echocardiography assessment revealed a 48 × 33 mm isoechoic mass in the right atrium, which had a relatively uniform shape and was tightly attached to the right atrial wall, exhibiting observable mobility. Color Doppler flow imaging analysis failed to detect any blood flow signals into the mass (Fig. 1A). Furthermore, cardiac computed tomography (CT) perfusion imaging demonstrated a homogeneous, low-density mass closely attached to the right atrial wall, exhibiting a CT value of ≈−114 HU, measuring 6.0 × 4.1 cm in size, and possessing a distinct boundary. No enhancement was observed within the mass (Fig. 1B and Supplemental Video S1, Supplemental Digital Content, http://links.lww.com/MD/O325). Preoperative evaluations, including coronary CT angiography and electrocardiogram examinations, indicated no evidence of myocardial ischemia. In the procedure, cardiopulmonary bypass was initiated by cannulating the ascending aorta and superior vena cava. Given concerns about the potential for the guidewire to enter the right atrium and entangle the tumor, or for venous cannulation to puncture the tumor leading to detachment, the decision was made to insert the inferior vena cava cannula proximal to the diaphragmatic plane of the right atrium. This approach facilitated tumor resection with a beating heart. The tumor, measured to be ≈5 × 6 cm^2^ in size, featured a yellow color, a soft texture, and was completely encapsulated (Supplemental Video S2, Supplemental Digital Content, http://links.lww.com/MD/O326). The tumor’s base was anchored to the anterolateral, unsupported wall of the right atrium, displaying an attachment width of roughly 3 × 4 cm^2^ (Fig. 2A–C). In addition, the portion of the right atrial wall adhering to the tumor was excised, and a bovine pericardial patch was tailored into an oval configuration, measuring 3 × 4 cm^2^, for the purpose of reconstructing the defect. Upon histopathological examination, it was ascertained that the tumor comprised numerous adipocytes (Fig. 2D), whereas the borders of the atrial wall adjacent to the tumor were devoid of adipocytes. Following the surgery, on the 66th postoperative day, the patient’s palpitation and fatigue had subsided, and echocardiography confirmed the absence of residual tumor, with only the pericardial patch evident (Supplemental Video S3, Supplemental Digital Content, http://links.lww.com/MD/O327).

## 3. Discussion

We report a case of a 52-year-old female with a large, well-circumscribed lipoma in the right atrium, highlighting the diagnostic and therapeutic strategies employed. The etiology of cardiac lipoma remains unclear, with current research focused on genetic mutations and abnormal myocardial cell proliferation. Recent study^[7]^ proposes potential link between cardiac lipomas and specific genetic alterations, including translocation of chromosome 12q13-15, gene deletion of 13q, and recombination of genes located on 6q21-23. Furthermore, Rainer et al^[3]^ has put forward a hypothesis suggesting that cardiac lipoma may originate in the connective tissue adjacent to the Sondergaard recess, subsequently infiltrating the right atrium through the atrial wall. This hypothesis is grounded on 4 key considerations: first, the repetitive contractile activity of the heart; second, the thin, compliant nature of the right atrial wall; third, the firm consistency of lipomas; and fourth, the progressive enlargement of these tumors over time. Unfortunately, we did not conduct any genetic analysis on the lipoma in this case.

The clinical presentation of cardiac lipomas exhibits a spectrum that is influenced by factors such as tumor size, anatomical location, and rate of growth. Individuals harboring small tumors remain asymptomatic. Conversely, those with large or rapidly progressive tumors may experience symptoms including chest pain, dyspnea, palpitations, and so forth.^[8]^ These manifestations may arise from the compression of the heart, adjacent blood vessels, or nerves by the lipoma. In specific instances, the detachment of tumor masses from subendocardial lipomas can lead to embolization. Myocardial lipomas have the potential to induce a range of arrhythmias and syncope, whereas subepicardial lipomas may elicit chest pain as a result of the pericardial compression of the coronary artery.^[9]^

Ultrasound serves as a primary diagnostic tool for cardiac lipoma due to its ease of use and accessibility. However, its relatively low resolution when imaging adipose tissue can hinder specificity. In cases where magnetic resonance imaging (MRI) is not feasible or ultrasound findings are inconclusive, CT scanning emerges as a viable alternative and complementary diagnostic method, given its high resolution and rapid imaging capabilities.^[10]^ MRI boasts high sensitivity and specificity in diagnosing cardiac lipoma, benefiting from advanced imaging techniques such as T1-weighted, T2-weighted, fat suppression and water suppression sequences. These features are invaluable for definitive diagnosis and differential diagnosis of lipomas.^[11]^ In the present case, echocardiography revealed no significant blood flow signals within the active mass, and cardiac CT angiography perfusion imaging clearly delineated the tumor boundaries without enhancement on contrast-enhanced scans. Although an MRI examination would have offered more detailed visualization of the tumor’s blood supply, the available imaging data were sufficient to make a confident diagnosis.

Asymptomatic patients with small tumors typically undergo regular observation and follow-up to monitor any changes in tumor size or the emergence of symptoms. However, patients with larger tumors or those exhibiting overt symptoms generally require a more proactive approach, with surgical intervention being strongly recommended. The median sternotomy technique is a well-recognized surgical method that offers extensive exposure of the heart and adjacent critical structures, allowing the surgeon to have a clear and unobstructed view of the operative area. In the presented case, the patient had a large tumor with a broad base in the right atrium, posing a significant challenge for surgeons. The median sternotomy approach enabled the surgeon to accurately assess the tumor’s dimensions and boundaries intraoperatively. This surgical access provided ample operating room for the surgeon to achieve a thorough tumor resection, thereby guaranteeing the precision and safety of the procedure. Following successful resection, a defect in the right atrium’s free wall was identified, necessitating a patch repair and reconstructive surgery. This critical step was essential for maintaining proper blood flow from the right atrium into the right ventricle, thereby preserving normal circulatory function. Intraoperative transesophageal echocardiography played a crucial role in real-time monitoring of cardiac function, providing valuable insights into cardiac rebeating patterns, chamber size, valve activity, and ventricular wall motion following posttumor resection, guiding surgical decisions in areas that may be challenging to visualize directly. However, in this particular case, the tumor’s location in the anterior wall of the right atrium minimized the need for intraoperative transesophageal echocardiography. Upon incision of the right atrium, the surgeon had a clear view of the tumor’s entirety, its attachment site, and its relationship with adjacent structures. For a patient with cardiac lipomas invading the right atrial free wall, preoperative anatomical evaluation is crucial to prevent over-resection, damage to the lateral bundle branch, and subsequent postoperative cardiac dysfunction or arrhythmia. Resection of tumors adjacent to the coronary sinus poses a risk of atrioventricular node or bundle damage, potentially leading to heart block. Therefore, electrocardiogram monitoring is essential during resection without cardiac arrest. Any abnormalities should prompt the suspension of the procedure or the insertion of a pacemaker. Deep incisions near the right atrioventricular groove should be avoided to protect the right coronary artery. Similarly, during the resection of tumors attached to the tricuspid valve, care must be taken to minimize valve damage. If valve damage does occur, prompt repair or replacement is necessary. In this patient, a defect was found on the right atrial wall postresection. Fortunately, after repair with a bovine pericardial patch, no conduction block occurred, and the patient had a favorable outcome.

## 4. Conclusion

Surgical intervention is indicated for symptomatic or large cardiac lipomas. In situations where significant defects are present in the atrial or ventricular wall, the use of artificial materials for repair or reconstruction becomes necessary to ensure optimal outcomes.

## Author contributions

**Conceptualization:** Zhi Wen.

**Data curation:** Zhi Wen.

**Writing – original draft:** Zhi Wen.

**Writing – review & editing:** Zhi Wen, Changxue Wu.

**Project administration:** Zheng Wang.

**Supervision:** Zheng Wang.

**Formal analysis:** Jian Guo.

**Software:** Jian Guo.

**Methodology:** Changxue Wu.

## Supplementary Material



## References

[1] Jayaprakash S. Clinical presentations, diagnosis, and management of arrhythmias associated with cardiac tumors. J Arrhythm. 2018;34:384–93. 30167009 10.1002/joa3.12030 PMC6111472

[2] Nishi H Mitsuno M Ryomoto M Hao H Hirota S Miyamoto Y. Giant cardiac lipoma in the ventricular septum involving the tricuspid valve. Ann Thorac Surg. 2009;88:1337–9. 19766837 10.1016/j.athoracsur.2009.02.052

[3] Rainer WG Bailey DJ Hollis HW Jr. Giant cardiac lipoma: refined hypothesis proposes invagination from extracardiac to intracardiac sites. Tex Heart Inst J. 2016;43:461–4. 27777537 10.14503/THIJ-15-5342 PMC5067047

[4] Reece IJ Cooley DA Frazier OH Hallman GL Powers PL Montero CG. Cardiac tumors. Clinical spectrum and prognosis of lesions other than classical benign myxoma in 20 patients. J Thorac Cardiovasc Surg. 1984;88:439–46. 6381889

[5] Li FP Wang XF Xiao J Xiao YB. Myocardial lipomatous infiltration of the left ventricular wall. J Card Surg. 2010;25:513–5. 20331480 10.1111/j.1540-8191.2010.01021.x

[6] Li D Wang W Zhu Z Wang Y Xu R Liu K. Cardiac lipoma in the interventricular septum: a case report. J Cardiothorac Surg. 2015;10:69. 25957090 10.1186/s13019-015-0275-0 PMC4431042

[7] Schrepfer S Deuse T Detter C. Successful resection of a symptomatic right ventricular lipoma. Ann Thorac Surg. 2003;76:1305–7. 14530040 10.1016/s0003-4975(03)00523-x

[8] Arhold S Elashry MI Klymiuk MC Geburek F. Investigation of stemness and multipotency of equine adipose-derived mesenchymal stem cells (ASCs) from different fat sources in comparison with lipoma. Stem Cell Res Ther. 2019;10:309. 31640774 10.1186/s13287-019-1429-0 PMC6805636

[9] Wu XQ Yang XY Li Y Wang XH Tong Q. Progress in the clinical diagnosis and treatment of cardiac lipoma. (In Chinese). J Shandong Med. 2018;58:109–12.

[10] Moskovitch G Chabbert V Escourrou G. Cardiac tumors: CT and MR imaging features. J Radiol. 2010;91:857–77. 20814374 10.1016/s0221-0363(10)70128-1

[11] Sun XF Liu GY Kim H Sun WY. Left ventricular lipoma resected using thoracoscope-assisted limited sternotomy: a case report and literature review. Medicine (Baltimore). 2018;97:e11436. 30075509 10.1097/MD.0000000000011436 PMC6081152

